# Musculoskeletal pain patterns and association between dizziness symptoms and pain in patients with long term dizziness – a cross-sectional study

**DOI:** 10.1186/s12891-023-06279-z

**Published:** 2023-03-08

**Authors:** Unni Moen, Mari Kalland Knapstad, Kjersti Thulin Wilhelmsen, Frederik Kragerud Goplen, Stein Helge Glad Nordahl, Jan Erik Berge, Bård Natvig, Dara Meldrum, Liv Heide Magnussen

**Affiliations:** 1 Department of Health and Functioning, Western Norway University of Applied Sciences, Bergen, Norway; 2 Norwegian National Advisory Unit On Vestibular Disorders, Haukeland University Hospital, Bergen, Norway; 3 Department of Otorhinolaryngology & Head and Neck Surgery, Haukeland University Hospital, Bergen, Norway; 4 Department of Clinical Medicine, University of Bergen, Bergen, Norway; 5 Department of General Practice, University of Oslo, Oslo, Norway; 6 School of Medicine, Trinity College Dublin, Dublin, Ireland

**Keywords:** Dizziness, Vertigo, Musculoskeletal pain

## Abstract

**Background:**

The impact of long-term dizziness is considerable both on the personal level and in society and may lead to self-imposed restrictions in daily activities and social relations due to fear of triggering the symptoms. Musculoskeletal complaints seem to be common in persons with dizziness, but studies addressing these complaints as a widespread occurrence, are scarce. This study aimed to examine the occurrence of widespread pain in patients with long-term dizziness and investigate the associations between pain and dizziness symptoms. Further, to explore whether diagnostic belonging is related to the occurrence of pain.

**Methods:**

This cross-sectional study was conducted in an otorhinolaryngology clinic and included 150 patients with persistent dizziness. The patients were categorized into three groups: episodic vestibular syndromes, chronic vestibular syndromes, and non-vestibular group. The patients completed questionnaires on dizziness symptoms, catastrophic thinking, and musculoskeletal pain when entering the study. Descriptive statistics were used to describe the population, and associations between pain and dizziness were investigated by linear regression.

**Results:**

Pain was reported by 94.5% of the patients. A significantly higher prevalence of pain was reported in all the ten pain sites examined compared to the general population. Number of pain sites and pain intensity were associated with the dizziness severity. Number of pain sites was also associated with dizziness-related handicap, but not with catastrophic thinking. There was no association between pain intensity and dizziness-related handicap or catastrophic thinking. Pain was equally distributed in the diagnostic groups.

**Conclusion:**

Patients with long-term dizziness have a considerably higher prevalence of pain and number of pain sites than the general population. Pain co-exists with dizziness and is associated with dizziness severity. These findings may indicate that pain should be systematically assessed and treated in patients with persisting dizziness.

**Supplementary Information:**

The online version contains supplementary material available at 10.1186/s12891-023-06279-z.

## Background

Dizziness is a common complaint in the general population, with an estimated lifetime prevalence of 15 – 35% [1–3]. The burden of dizziness impacts both social life and society and is associated with increased healthcare use and sick leave [1, 4, 5]. Physical and social activities are limited from fear of triggering dizziness, resulting in a restricted movement pattern and increased muscular tension [6, 7]. Falls, mental health consequences, reduced quality of life as well as musculoskeletal pain are frequently reported [8–10].

Musculoskeletal complaints are common, also among patients with dizziness [10–12], but previous studies have primarily addressed pain in the neck area [13–15]. However, neck pain is also found to be part of a widespread pain pattern in the general population [16], and this could probably apply to dizziness populations as well. A recent systematic review found that pain in other body parts is also evident in patients with dizziness [17], and dizziness is further found to be associated with local as well as widespread pain [18]. It is possible to speculate that both dizziness and pain could have a mutual preserving effect on each other as both symptoms could result in a more rigid movement pattern, leading to increased muscular tension and reduced recovery from dizziness [6, 7]. Catastrophic thinking occurs frequently among patients experiencing persistent dizziness [19] as well as among patients with chronic pain [20]. This involves fear and worry about expected or actual symptoms and may also contribute to increased symptom severity and negative clinical outcome.

Studies that systematically examine musculoskeletal pain in patients with dizziness are scarce. Thus, the present study aimed to examine the prevalence, intensity and, distribution of musculoskeletal pain in patients presenting with persistent dizziness in an otorhinolaryngology clinic. Further, we wanted to investigate possible associations between dizziness symptoms and musculoskeletal pain.

## Method

### Design and settings

A cross-sectional study investigating pain in patients with long-term dizziness was conducted by recruiting consecutive outpatients examined in a specialized Balance Clinic at an otolaryngology clinic at a University hospital. The patients were referred from primary or specialist care due to dizziness or balance problems.

### Subjects

**Fig. 1:**
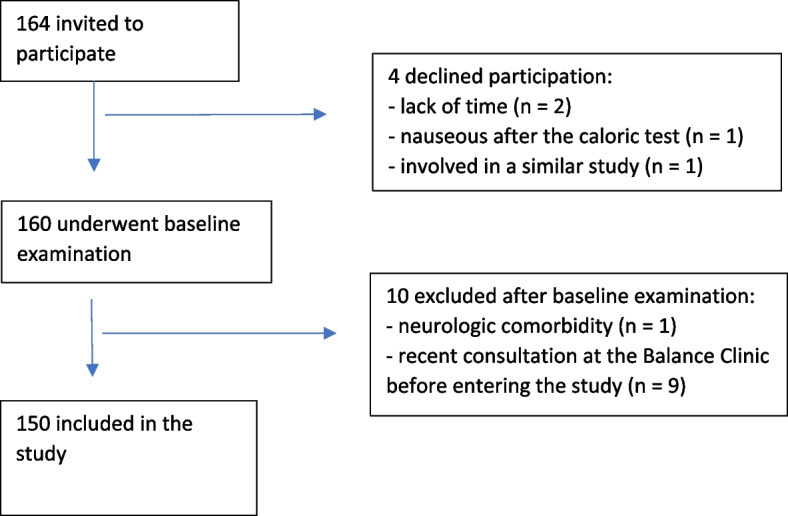
The flow diagram details the process of including patients

Patients aged 18–67, were eligible for inclusion if dizziness had lasted at least three months. Inclusion period was between August 2020 and January 2022 and a total of 164 patients were invited to participate in the study. Hospitalized patients and patients with neurological disorders (e.g. Multiple Sclerosis, Parkinson, stroke) or other serious comorbidities (e.g. amputations, alcoholism, ongoing chemotherapy) that potentially could affect physical functioning were excluded. Patients with vestibular schwannomas, divers investigated for neuro-otologic disorders, and patients from other health authority regions were also excluded. The patients had to have sufficient knowledge of Norwegian to fill in the questionnaires. Of the 164 eligible patients, four declined to participate, ten were later excluded, leaving 150 included patients (Fig. 1).

### Data collection

#### Demographic variables

Age, gender, employment status, and duration of dizziness were registered.

#### Diagnoses

Diagnoses were set retrospectively by three experienced otorhinolaryngology specialists blinded to each other (F.G, S.H.G.N, J.E.B), according to the International Classification of Diseases (ICD-11). Discrepancy between the specialist were further resolved by consensus. If the patient had several diagnoses, the one determined as the main diagnosis was included in the analysis. The diagnoses were further categorized into three categories corresponding to the ICD-11 coding system: *Episodic vestibular syndrome* (AB31 codes) including Benign Paroxysmal Positional Vertigo (BPPV), vestibular migraine, Ménière disease, and unspecified episodic syndromes; *Chronic vestibular syndrome* (AB32 codes) including Persistent Postural-Perceptual Dizziness (PPPD), vestibulopathy and specified or unspecified chronic syndromes, and *other* (MB23, MB44) including panic attack and abnormalities of gait and mobility (Table 1). The overall distribution of diagnoses is listed in Additional file 1.

**Table 1:** Descriptive statistics of 150 patients with long-term dizziness according to the number of pain sites and pain intensity (dependent variables) across symptoms of dizziness (independent variables)

Variables	Total	Number of pain sites (CI/SD)	Pain intensity (NRS) (CI/SD)
Total sample n (%)	150 (100)	4.5 (2.5)	4.0 (2.3)
Female	97 (65)	4.9 (4.4 – 5.4)	4.3 (3.9 – 4.8)
Male	53 (35)	3.7 (3.0 – 4.3)	3.5 (2.8 – 4.1)
Age mean (SD)	46.5 (12.7)		
Diagnostic categories (ICD-11) n (%)			
Episodic vestibular syndrome	97 (64.7)	4.4 (2.4)	4.2 (2.3)
Chronic vestibular syndrome	49 (32.7)	4.7 (2.7)	3.8 (2.4)
Other	4 (2.7)	3.5 (3.4)	3.3 (2.5)
Sick leave /disability benefits n (%)			
*No sick leave or disability benefits:*	85 (56.6)	4.1 (2.5)	4.0 (2.3)
*Part-time sick leave*	20 (13.3)	5.0 (2.8)	3.5 (2.5)
*Full-time sick leave*	29 (19.3)	4.3 (2.5)	3.8 (2.3)
*Disability benefits*	16 (10.7)	5.8 (2.0)	5.4 (2.0)
Dizziness duration months, median (IQR)	21.5 (45.5)		
VSS-SF Total mean (SD)	17.1 (9.5)		
*VSS-SF – autonomic-anxiety*	7.3 (5.0)		
*VSS-SF – vertigo-balance*	9.8 (5.8)		
VSS-SF < 12 n (%)	45 (31)	3.6 (2.8 – 4.3)	3.4 (2.7 – 4.2)
VSS-SF ≥ 12 n (%)	101 (69)	4.9 (4.4 – 5.3)	4.3 (3.9 – 4.7)
DHI total mean (SD)	38 (20.1)		
DHI < 30 n (%)	51 (35)	4.1 (3.4 – 4.8)	3.6 (2.9 – 4.3)
DHI ≥ 30 n (%)	95 (65)	4.7 (4.1 – 5.2)	4.3 (3.8 – 4.7)
DCS total mean (SD)	21.2 (11.9)		

*Abbreviations*: *CI, 95%* Confidence Interval, *DCS* Dizziness Catastrophizing Scale, *DHI* Dizziness Handicap Inventory, *ICD-11* International Classification of Diseases 11^th^ Revision, *IQR* Interquartile range, *NPS* Number of pain sites, *NRS* Numeric rating scale of pain, *SD* Standard deviation, *VSS-SF* Vertigo Symptom Scale -short form

#### Patient-reported outcome measures

The Dizziness Handicap Inventory (DHI) [21] quantifies the impact of dizziness on daily life. The 25-item questionnaire reflects physical, functional, and emotional aspects of dizziness. The response categories “yes” (4 points), “sometimes” (2 points), and “no” (0 points) present a total score from 0–100. Scores 0—29 represent a mild dizziness handicap, 30 – 60 moderate handicap, and > 60 severe handicap [22].

The Vertigo Symptom Scale – Short Form (VSS-SF) has satisfactory internal consistency and test–retest reliability and measures the frequency and severity of dizziness symptoms [23]. VSS-SF contains 15 items, 8 related to vertigo-balance and 7 related to autonomic-anxiety symptoms. Each item is scored on a 5-point scale (0–4), giving a total score of 60, where higher scores indicate higher severity. A total score of ≥ 12 points indicates severe dizziness [24].

The Standardized Nordic Pain Questionnaire (SNQ) traces the localization of musculoskeletal pain or discomfort by the following question: “Do musculoskeletal troubles occur in a given population, and if so, in what part of the body are they localized?” [25, 26]. The respondent is asked to identify pain or discomfort in 10 different body sites: head, neck, shoulders, elbows, wrist/hands, upper back, lower back, hips, knees, and ankle/feet, during the last 7 days. A mannequin drawing illustrates the delimitation of the 10 different body sites. Localization and number of pain sites (NPS) are registered.

Pain intensity during the last seven days was reported by an 11-point (0–10) Numeric Rating Scale (NRS) where 0 equals “no pain at all” and 10 equals “worst imaginable pain”. NRS is reported to be valid and reliable [27, 28]. NRS ≤ 5 is considered mild pain-related interference 6 and 7: moderate interference, and ≥ 8, severe interference with functioning [29].

Dizziness Catastrophizing Scale (DCS) measures dizziness-related catastrophizing. It comprises 13 self-reported items, scored on a 5-point (0–4) Likert-type of scale where 0 equals “not at all” and 4 equals “all the time” [19]. The total score is 52 and higher scores indicate increased presence of catastrophizing. DCS has demonstrated good validity and reliability [19]. Validation of the Norwegian version is ongoing.

### Statistical analysis

Descriptive statistics were used for demographic data, test scores, prevalence of pain, and pain pattern distribution; and are presented as mean, median, and frequency distribution as appropriate. Continuous variables were assessed and deemed normally distributed using visual inspections of histograms and qq-plots.

Linear regression analysis was used to estimate the association between pain (NPS and NRS) as dependent variables and dizziness (VSS-SF, DHI, DCS) as independent variables, and was examined in crude and adjusted models. Confounding variables (age and sex) were included in the adjusted regression models. The alpha level was set to 0.05. Of the 150 included participants, four had missing data, which was deemed insignificant to the results. The statistical software package Stata 17 was used for the data analysis.

### Ethics

The study was approved by the Regional Ethical Committee (REK) (REK 2019/6849) and Norwegian Centre for Research Data (NSD), and committed to the criteria laid in the current (2013) Declaration of Helsinki (www.wma.net) and the personal data were administered following the standards of the General Data Protection Regulation (GDPR). To provide transparency, the study was registered in the Clinical Trials database (NCT04241822 27/01/2020) prior to the data collection. The authors declare no conflict of interest.

## Results

### Characteristics of the sample and prevalence of symptoms

In total 150 patients were included in the study, 65% women, mean age 46.5 (range 22 – 67) years. Median duration of dizziness was 21.5 (range 3—509) months. Sick leave or disability was reported by 43%, of those, 60% stated dizziness as the cause. Episodic vestibular syndromes were diagnosed in 65% of the patients, while 33% were diagnosed with a chronic vestibular syndrome. Four patients received a non-vestibular diagnosis. Average number of pain sites was 4.5. Higher intensity of pain and higher number of pain sites were seen among patients on disability benefits and among those patients who reported severe dizziness (VSS-SF ≥ 12) and moderate to severe dizziness-related handicap (DHI ≥ 30). Females tended to have a higher number of pain sites and higher pain intensity than men. The clinical and demographic characteristics are listed in Table 1.

**Fig. 2:**
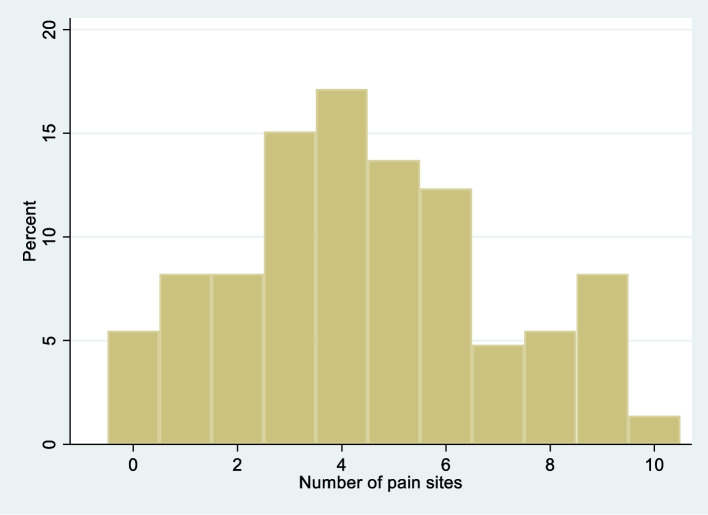
Frequency distribution of the total number of pain sites reported

**Fig. 3:**
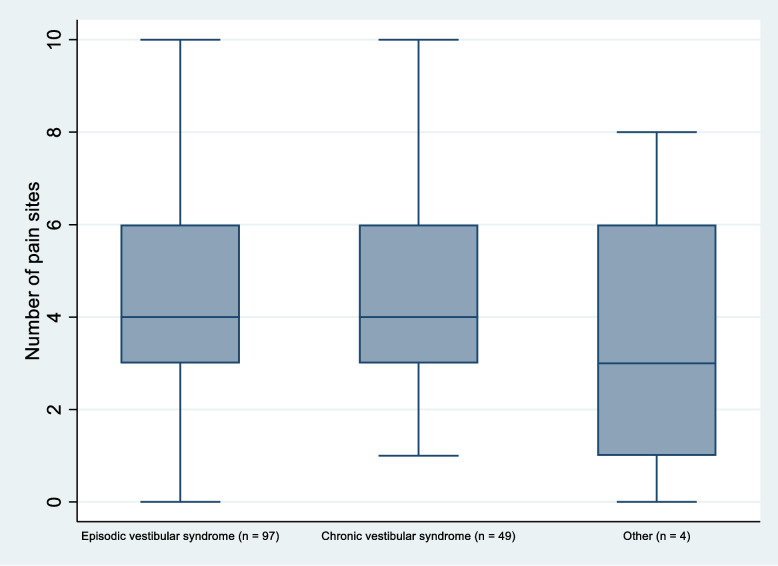
The distribution and variation of the number of pain sites across the three diagnostic categories

**Fig. 4:**
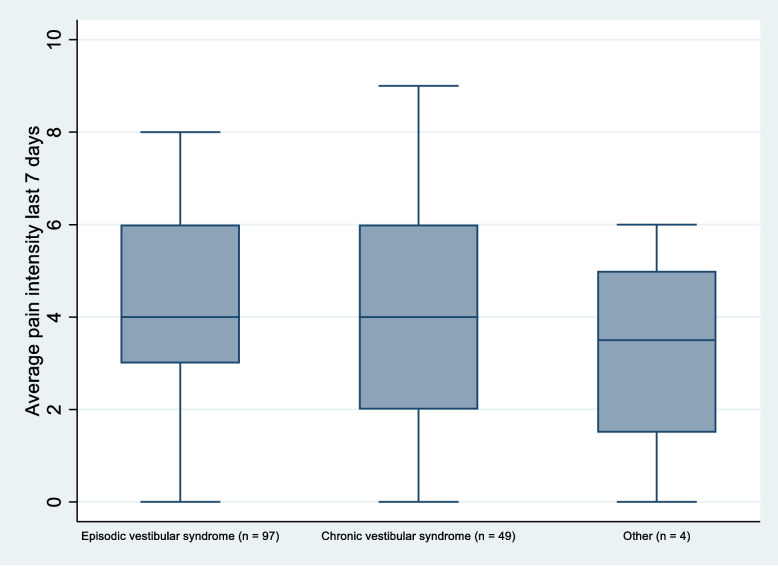
The distribution and variation of pain intensity across the three diagnostic categories

The majority (94.5%) of the patients reported pain or discomfort in at least one body site during the past week. Figure 2 shows the distribution of NPS. Pain in the head and neck was most frequently reported, followed by lower back, shoulders, upper back, hips, wrist/hands, knees, ankle/feet, and elbows. The distribution of pain areas (Table 2), frequency/distribution of number of pain sites (Fig. 3) and pain intensity (Fig. 4) did not seem to differ between the diagnostic categories.

**Table 2:** Localization of pain or discomfort according to SNQ in the total group (*n* = 146) and by diagnostic categories

**Pain site**	**Total** (*n* = 146)	**Chronic** (*n* = 49)	**Episodic** (*n* = 97)	**Other** (*n* = 4)
Head n (%)	110 (75.3)	39 (79.6)	68 (70.1)	3 (75)
Neck n (%)	102 (69.9)	37 (75.5)	63 (64.9)	2 (50)
Lower back n (%)	91 (62.3)	30 (61.2)	58 (59.8)	3 (75)
Shoulders n (%)	90 (61.6)	30 (61.2)	58 (59.8)	4 (100)
Upper back n (%)	60 (41.1)	21 (42.9)	38 (39.2)	1 (25)
Hips n (%)	49 (33.6)	18 (36.7)	30 (30.9)	1 (25)
Wrist/hands n (%)	47 (32.2)	19 (38.8)	27 (27.8)	1 (25)
Knees n (%)	46 (31.5)	14 (28.6)	32 (33.0)	0 ( 0)
Ankle/Feet n (%)	39 (26.7)	15 (30.6)	24 (24.7)	0 ( 0)
Elbows n (%)	17 (11.6)	8 (16.3)	8 ( 8.2)	1 (25)

### Associations between dizziness and musculoskeletal symptoms

Linear regression analysis showed a statistically significant positive association between the number of pain sites (NPS) dizziness severity (VSS-SF) and dizziness-related handicap (DHI) (Table 3). There was no association between NPS and catastrophic thinking (DCS). There was a statistically significant association between pain intensity (NRS) and dizziness severity (VSS-SF), but not between NRS and DHI or DCS (Table 4).

**Table 3:** Association between the number of pain sites as the dependent variable and dizziness symptoms, dizziness-related handicap, or dizziness catastrophizing thoughts as independent variables, examined with linear regression analysis. Adjusted for age and sex (*n* = 150)

Number of pain sites				
*Variables*	Coef	CI	*P*	R^2^
**VSS-SF total**				
Crude	.090	.048-.133	.000	.112
Adjusted	.085	.038-.131	.000	.139
**DHI total**				
Crude	.029	.008-.050	.007	.051
Adjusted	.024	.002-.046	.033	.085
**DCS total**				
Crude	.028	-.007-.062	.118	.017
Adjusted	.032	-.004-.067	.082	.077

*Abbreviations*: *CI* Confidence intervals, *Coef* Coefficient, *DCS* Dizziness Catastrophizing Scale, *DHI* Dizziness Handicap Inventory, *p* p-value, *R*^*2*^ R-squared, *VSS-SF* Vertigo Symptom Scale – short form

*p* < .05

**Table 4:** Association between pain intensity as the dependent variable, and dizziness symptoms, dizziness-related handicap, or dizziness catastrophizing thoughts as independent variables, examined with linear regression analysis. Adjusted for age and sex (*n* = 150)

Pain intensity (NRS)				
*Variables*	Coef	CI	*P*	R^2^
**VSS-SF total**				
Crude	.065	.024—.105	.002	.067
Adjusted	.069	.025—.113	.002	.097
**DHI total**				
Crude	.019	-.000—.039	.053	.027
Adjusted	.018	-.003—.038	.087	.055
**DCS total**				
Crude	.022	-.011—.054	.188	.012
Adjusted	.030	-.003—.063	.072	.060

*Abbreviations*: *CI* Confidence intervals, *Coef* Coefficient, *DCS* Dizziness Catastrophizing Scale, *DHI* Dizziness Handicap Inventory, *NRS* Numeric Rating Scale, *p* p-value, *R*^*2*^ R-squared, *VSS-SF*, Vertigo Symptom Scale – short form

*p* < .05

## Discussion

This study found that most of the patients with dizziness reported pain (94.5%), with an average of 4.5 number of pain sites. Number of pain sites and pain intensity were associated with the dizziness severity. Number of pain sites was also associated with dizziness-related handicap.

The high prevalence of pain reported in our study is in line with other studies on patients with dizziness [10, 17, 18]. Despite that estimates of pain prevalence may vary between studies due to differences in methodology, definitions, and context, the prevalence in the current study was substantially higher than in the general population in Norway (94.5% versus 25%) [30, 31]. In a Norwegian population study counting the same NPS [26], 70% of the responders reported pain or discomfort from at least one body site during the last week versus 94.5% in our study. It has been suggested that the number of pain sites is strongly associated with reduced general health, and non-musculoskeletal symptoms (e.g. dizziness) and is also reported to be a predictor of future disability [26, 32, 33]. The average number of pain sites during the last week in our participants was 4.5 compared to 2.3 in the general population [26]. Further, 45.9% of our patients reported pain from five or more sites and 19.9% reported pain from seven or more sites (Fig. 2), which is considerably more compared to the general population who reported 17 and 7% respectively [32].

The high prevalence of pain in our patients may therefore indicate a greater health burden and risk of future health problems. There seemed to be no difference in pain across the three diagnostic categories. Pain, including the number of pain sites, should therefore be taken into consideration in the examination and treatment of all patients with persistent dizziness.

The pattern of pain localization, where the neck, back, and shoulders are the most reported pain sites, is similar to what reported in other studies on patients with dizziness [17, 34] and in the general population [26, 32]. However, the frequency was markedly higher in our study. Regarding musculoskeletal pain, neck pain has been the main concern in studies regarding patients with dizziness. This is probably due to the known neurophysiological connections between the vestibular and visual systems, and cervical spine structures [35, 36]. Persons with dizziness may avoid head movement out of fear of triggering dizziness and adopt a compensating postural strategy to maintain balance. This may lead to a more rigid movement pattern and increased muscular tension.

The regression analysis showed a statistically significant association between number of pain sites and dizziness severity (VSS-SF) (*p* = *0.00*) as well as dizziness-related handicap (DHI) (*p* = *0.03*). The number of pain sites may therefore be an indicator of a greater symptom-burden of dizziness and could perhaps influence dizziness symptoms. This is consistent with a previous study from Malmström, Magnusson [34] who found that patients with dizziness are likely to experience pain in the neck, shoulders and/or back (NSB).

We hypothesized that the number of pain sites and pain intensity would be associated with the degree of handicap and catastrophic thoughts due to dizziness. However, we found no association between pain intensity and dizziness-related handicap (DHI) as the confidence intervals contains the value of zero, although there was a tendency for higher pain intensity and NPS in the group with DHI scores ≥ 30 (Table 1). This is in line with a previous study by Cuenca-Martinez, Bartrina-Rodriguez [11] which examined the correlation between dizziness-related handicap and pain pressure threshold. The intensity of pain was however not different in our population than in the general population [31]. As catastrophizing contributes to an overall clinical burden, we hypothesized that catastrophic thinking on dizziness would be associated with the perception of pain. There was however a lack of association between both pain intensity or the number of pain sites and catastrophic thoughts about dizziness (DCS), with p-values > 0.05 and the confidence intervals contains zero. This may be because these questionnaires are developed to capture how dizziness and catastrophic thinking interfere with daily life without considering pain as an element related to dizziness.

### Strengths and limitations

The population in this study was patients referred to a specialized otolaryngology clinic due to dizziness. The study population may differ from other persons with persistent dizziness treated in primary care, as they are referred to a specialized clinic due to the severity of complaints, introducing a selection bias. Selection bias may also exist due to the Covid-19 pandemic, as some patients may for this reason have canceled their appointments at the hospital. However, as only four out of 164 patients refused to participate in the study, it is reasonable to assume that the results are representative of the patients referred to the balance clinic in this period.

It is challenging to assess patients with complex conditions and the included outcomes could have shortcomings in capturing central aspects of both dizziness and pain. The SNQ questionnaire does not distinguish between musculoskeletal pain and other types of pain such as neuropathic pain. Many patients are, however, unable to distinguish between the different types or etiology of their pain, therefore, we cannot exclude that some of the reported pain may be of other causes such as sciatica, carpal tunnel syndrome, or organ-related pain. When comparing our study population to the general population, the same measuring tools were used making the comparisons more reliable. A causality between musculoskeletal complaints and dizziness was not possible to establish due to the cross-sectional design.

Future research should examine possible predictive relationships between dizziness and musculoskeletal symptoms. Another important aspect would be to address the patients’ perceptions of how pain and dizziness influence each other, and how both interfere with daily life, through a qualitative study. Both types of studies would provide insights that potentially could lead us towards more tailored rehabilitation for these patients.

### Clinical relevance

The prognosis of persistent dizziness is poor in many cases [8, 37], hence there is a need to look at other potential aspects that may prevent successful recovery. This study may contribute to a better understanding of a population in a need of interventions that goes beyond traditional vestibular rehabilitation [38]. It is well known that psychological components such as anxiety and depression are associated with persisting dizziness and in many cases a natural part of the examination of these patients. Our findings raise the question whether musculoskeletal pain also should be assessed more systematically. This is, according to our experience, only cursory done in clinical practice today. It should be undertaken routinely to capure the overall picture of the patients problems. Patients may be entangled in a “vicious circle” where dizziness and pain reciprocally aggravate and sustain each other – and possibly reinforced by anxiety, adding to the complexity. In such case a single diagnose or measures is less useful. Assessing and treating the musculoskeletal symptoms as well the vestibular and potential psychological aspects, could break the circle in several ways simultaneously and thereby influence rehabilitation positively.

## Conclusion

The prevalence of pain and number of pain sites are considerably higher in our study sample of patients compared to the general population. Both number of pain sites and pain intensity were associated with dizziness severity. The localization of pain sites and the pain intensity are however in line with what is seen in the general population in Norway. The diagnostic category does not seem to play a role when dizziness persists, as the number of pain sites and pain intensity were similar between groups.

These findings may indicate a greater health burden and an increased risk of future health problems in patients with persistent dizziness. Musculoskeletal pain, including the number of pain sites, should therefore be taken into consideration in the examination and treatment of these patients.

## Supplementary Information

**Additional file 1. **The distribution of diagnoses in the study sample, according to the ICD-11 system.

## Data Availability

The datasets used and analyzed during the current study are available from the corresponding author on reasonable request.

## References

[1] Neuhauser HK. The epidemiology of dizziness and vertigo. In: Furman JM, Lempert T, editors. Handbook Clin Neurol. Elsevier; 2016;137:67–82. 10.1016/B978-0-444-63437-5.00005-4 27638063

[2] Hain TC. Epidemiology of dizziness. Chicago Dizziness and Hearing; 2021. Updated May 1, 2022. Available from: https://dizziness-and-balance.com/disorders/dizzy_epi.html.

[3] Murdin L, Schilder AGM (2015). Epidemiology of balance symptoms and disorders in the community: a systematic review. Otol Neurotol.

[4] Kovacs E, Wang X, Grill E (2019). Economic burden of vertigo: a systematic review. Heal Econ Rev.

[5] Benecke H, Agus S, Kuessner D, Goodall G, Strupp M (2013). The burden and impact of vertigo: findings from the REVERT patient registry. Front Neurol.

[6] Coelho Júnior AN, Gazzola JM, Gabilan YPL, Mazzetti KR, Perracini MR, Gananca FF (2010). Head and shoulder alignment among patietns with unilateral vestibular hypofunction. Braz J Phys Ther.

[7] Wilhelmsen KT, Kvåle A (2014). Examination and treatment of patients with unilateral vestibular damage, with focus on the musculoskeletal system: a case series. Phys Ther.

[8] Lahmann C, Henningsen P, Brandt T, Strupp M, Jahn K, Dieterich M (2015). Psychiatric comorbidity and psychosocial impairment among patients with vertigo and dizziness. Neuropsychiatry.

[9] Weidt S, Bruehl AB, Straumann D, Hegemann SCA, Krautstrunk G, Rufer M (2014). Health-related quality of life and emotional distress in patients with dizziness: a cross-sectional approach to disentangle their relationship. BMC Health Serv Res.

[10] Malmström EM, Hansson EE, Hafstrom A, Magnusson M, Fransson PA. Co-morbidities to vestibular impairments -Some concomitant disorders in young and older adults. Front Neurol. 2021;11:609928. 10.3389/fneur.2020.609928 PMC7873354 33584509

[11] Cuenca-Martinez F, Bartrina-Rodriguez I, Suso-Marti L, La Touche R, Ferrer-Pena R (2018). Association between somatosensory, motor and psychological variables by levels of disability in patients with cervicogenic dizziness. Somatosens Mot Res.

[12] Iglebekk W, Tjell C, Borenstein P (2013). Pain and other symptoms in patients with chronic benign paroxysmal positional vertigo (BPPV). Scand J Pain.

[13] Malmström E-M, Karlberg M, Melander A, Magnusson M, Moritz U (2007). Cervicogenic dizziness - musculoskeletal findings before and after treatment and long-term outcome. Disabil Rehabil.

[14] Knapstad MK, Goplen F, Skouen JS, Ask T, Nordahl SHG (2019). Symptom severity and quality of life in patients with concurrent neck pain and dizziness. Disabil Rehabil.

[15] Knapstad MK, Nordahl SHG, Goplen F (2019). Clinical characteristics in patients with cervicogenic dizziness: a systematic review. Health Sci Rep.

[16] Natvig B, Ihlebæk C, Grotle M, Brage S, Bruusgaard D (2010). Neck pain is often a part of widespread pain and is associated with reduced functioning. Spine (Phila Pa 1976).

[17] Moen U, Magnussen LH, Wilhelmsen KT, Goplen FK, Nordahl SHG, Meldrum D, Knapstad MK. Prevalence and distribution of musculoskeletal pain in patients with dizziness—a systematic review. Physiother Res Int. 2022;27:e1941. 10.1002/pri.1941 PMC9286866 35191148

[18] Gustavsen IØ, Wilhelmsen KT, Goode AP, Nordahl SHG, Goplen FK, Nilsen RM, Magnussen LH. Dizziness and physical health are associated with pain in dizzy patients—a cross-sectional study. Physiother Res Int. 2021;26(4):e1923. 10.1002/pri.1923 34585499

[19] Pothier DD, Shah P, Quilty L, Ozzoude M, Dillon WA, Rutka JA (2018). Association between catastrophizing and dizziness-related disability assessed with the dizziness catastrophizing scale. JAMA Otolaryngol Head Neck Surg.

[20] Sullivan MJL, Thorn B, Haythornthwaite JA, Keefe F, Martin M, Bradley LA (2001). Theoretical perspectives on the relation between catastrophizing and pain. Clin J Pain.

[21] Tamber AL, Wilhelmsen KT, Strand LI (2009). Measurement properties of the dizziness handicap Inventory by cross-sectional and longitudinal designs. Health Qual Life Outcomes.

[22] Whitney SL, Wrisley DM, Brown KE, Furman JM (2004). Is perception of handicap related to functional performance in persons with vestibular dysfunction?. Otol Neurotol.

[23] Wilhelmsen KT, Strand LI, Nordahl SHG, Eide GE, Ljunggren AE (2008). Psychometric properties of the vertigo symptom scale – short form. BMC Ear Nose Throat Disord.

[24] Yardley L, Donovan-Hall M, Smith HE, Walsh BM, Mullee M, Bronstein A (2004). Effectiveness of primary care-based vestibular rehabilitation for chronic dizziness. Ann Intern Med.

[25] Kuorinka I, Jonsson B, Kilbom A, Vinterberg H, Biering-Sørensen F, Andersson G (1987). Standardised Nordic questionnaires for the analysis of musculoskeletal symptoms. Appl Ergon.

[26] Tschudi-Madsen H, Kjeldsberg M, Natvig B, Ihlebaek C, Dalen I, Kamaleri Y (2011). A strong association between non-musculoskeletal symptoms and musculoskeletal pain symptoms: results from a population study. BMC Musculoskelet Disord.

[27] Ferreira-Valente MA, Pais-Ribeiro JL, Jensen MP (2011). Validity of four pain intensity rating scales. PAIN.

[28] Williamson A, Hoggart B (2005). Pain: a review of three commonly used pain rating scales. J Clin Nurs.

[29] Boonstra AM, Stewart RE, Köke AJA, Oosterwijk RFA, Swaan JL, Schreurs KM (2016). Cut-off points for mind, moderate and severe pain on the numeric rating scale for pain in patients with chronic musculoskeletal pain: variability and influence of sex and catastrophizing. Front Psychol.

[30] Kinge JM, Knudsen AK, Skirbekk V, Vollset SE (2015). Musculoskeletal disorders in Norway: prevalence of chronicity and use of primary and specialist health care services. BMC Musculoskel Disord.

[31] Rustøen T, Wahl AK, Hanestad BR, Lerdal A, Paul S, Miaskowski C (2004). Prevalence and characteristics of chronic pain in the general Norwegian population. Eur J Pain.

[32] Kamaleri Y, Natvig B, Ihlebaek C, Benth JS, Bruusgaard D (2008). Number of pain sites is associated with demographic, lifestyle, and health-related factors in the general population. Eur J Pain.

[33] Mose S, Kent P, Smith A, Andersen JH, Christiansen DH (2021). Number of musculoskeletal pain sites leads to increased long-term healthcare contacts and healthcare related costs - a Danish population-based cohort study. BMC Health Serv Res.

[34] Malmström E-M, Magnusson M, Holmberg J, Karlberg M, Fransson PA. Dizziness and localized pain are often concurrent in patients with balance or psychological disorders. Scand J Pain. 2019;20(2):353–62. 10.1515/sjpain-2019-0121 31881001

[35] Kristjansson E, Treleaven J (2009). Sensorimotor function and dizziness in neck pain: implications for assessment and management. J Orthop Sports Phys Ther.

[36] Treleaven J (2008). Sensorimotor disturbances in neck disorders affecting postural stability, head and eye movement control. Man Ther.

[37] Bösner S, Schwarm S, Grevenrath P, Schmidt L, Hörner K, Beidatsch D (2018). Prevalence, aetiologies and prognosis of the symptom dizziness in primary care – a systematic review. BMC Fam Pract.

[38] Hall CD, Herdman SJ, Whitney SL, Anson ER, Carender WJ, Hoppes CW (2022). Vestibular rehabilitation for peripheral vestibular Hypofunction: an updated clinical practice guideline From the Academy of Neurologic Physical Therapy of the American Physical Therapy Association. J Neurol Phys Ther.

