# Supplementary material for: Musculoskeletal pain patterns and association between dizziness symptoms and pain in patients with long term dizziness – a cross-sectional study
Source: BMC Musculoskelet Disord. 2023 Mar 8;24:173. doi: 10.1186/s12891-023-06279-z (PMC9992911; doi:10.1186/s12891-023-06279-z)
Supplement: Supplementary file 1 — Additional file 1. The distribution of diagnoses in the study sample, according to the ICD-11 system. [file 12891_2023_6279_MOESM1_ESM.docx]

Appendix 1: The distribution of diagnoses in the study sample, according to the ICD-11 system.

| Diagnoses | ICD-11 | n | % |
| --- | --- | --- | --- |
| Vestibular neuritis | AB30.0 | 1 | 0.7 |
| Ménières disease | AB31.0 | 16 | 10.7 |
| Vestibular migraine | AB31.1 | 21 | 14.0 |
| BPPV | AB31.2 | 24 | 16.0 |
| Superior canal dehiscence syndrome | AB31.3 | 2 | 1.3 |
| Disembarkment syndrome | AB31.4 | 1 | 0.7 |
| Other specified episodic vestibular syndrome | AB31.Y | 2 | 1.3 |
| Episodic vestibular syndrome, unspecified | AB31.Z | 31 | 20.7 |
| PPPD | AB32.0 | 34 | 22.7 |
| Chronic unilateral idiopathic vestibulopathy | AB32.1 | 1 | 0.7 |
| Persistent unilateral vestibulopathy after vestibular neuronitis | AB32.2 | 6 | 4.0 |
| Other specified chronic vestibular syndrome | AB32.Y | 4 | 2.7 |
| Chronic vestibular syndrome, unspecified | AB32.Z | 4 | 2.7 |
| Panic attack | MB23.H | 2 | 1.3 |
| Abnormalities of gait and mobility, unspecified | MB44.Z | 1 | 0.7 |
|  |  | 150 | 100 |

Abbrevations: BPPV, Bening Paryxosmal Positional Vertigo; PPPD, Persistent Postural-Perceptual Dizziness
